# RETRACTION: Efficacy of Subconjunctival Bevacizumab Injections before and after Surgical Excision in Preventing Pterygium Recurrence

**DOI:** 10.1155/joph/9895280

**Published:** 2026-10-16

**Authors:** Journal of Ophthalmology

RETRACTION: R. Nuzzi and F. Tridico, “Efficacy of Subconjunctival Bevacizumab Injections before and after Surgical Excision in Preventing Pterygium Recurrence,” *Journal of Ophthalmology*, 2017 (2017), https://doi.org/10.1155/2017/6824670.

The above article, published in print on May 18, 2017, has been retracted following the agreement of the journal’s Chief Editor, Yong Tao; and John Wiley & Sons Ltd.

This article has been retracted following a confirmation from the Italian Medicines Agency (AIFA), whose investigation revealed multiple concerns that compromise the integrity of the data presented in the article. These concerns include the following:

- The study described in the article was approved by an unaccredited ethics committee.
- The AIFA inspection could not locate any documentation that would validate that a legitimate clinical trial was carried out as described in the article.
- The data reported in the article could not be supported by the clinical records of the patients at the San Luigi Gonzaga University.

The authors were asked on multiple occasions to reply to these concerns, but no response was received. As a consequence, the journal and publisher no longer have confidence in the research described in the article and it is therefore retracted.

The authors were informed of this retraction but did not provide a response.

